# Tryptophan metabolism in psoriasis and its complications: Future opportunities

**DOI:** 10.1016/j.jare.2025.09.034

**Published:** 2025-09-20

**Authors:** Xiyuan He, Yueting Mo, Peixin Shi, Yini Xu, Mingmei Zhou, Ting Zhang

**Affiliations:** a Institute of Interdisciplinary Integrative Medicine Research, Shanghai University of Traditional Chinese Medicine, Shanghai 201203, China; b State Key Laboratory of Functions and Applications of Medicinal Plants, Guizhou Medical University, Guiyang 550014, China

**Keywords:** Psoriasis, Tryptophan, Psoriasis comorbidities, Metabolism, Treatment

## Abstract

Tryptophan (Trp) metabolism encompasses primary three pathways: the kynurenine pathway, the serotonin pathway, and the indole pathway. These pathways are intricately involved in the physiological and pathological processes underlying psoriasis, exerting broad regulatory effects on immune function, inflammation responses, keratinocytes proliferation, angiogenesis, and intestinal homeostasis. Key enzymes and metabolites within the Trp metabolic pathway have been implicated in the development and progression of psoriasis. Disruptions in Trp metabolism have been observed in psoriasis and its associated complications, with alterations in certain metabolite levels noted following improvements in disease symptoms. Emerging evidence indicates that targeted therapies modulating Trp metabolism have demonstrated efficacy in clinical applications, offering valuable insights for medication selection in psoriasis treatment. This review aims to summarize the most recent research findings regarding the role of Trp metabolism in psoriasis and its comorbidities, as well as the therapeutic potential of targeting Trp metabolic pathways.

## Highlights

• Tryptophan and its metabolites modulate diverse pathophysiology.
• Tryptophan pathway enzymes/metabolites drive psoriasis and comorbidities.
• Targeting these enzymes/metabolites offers a novel anti-psoriasis strategy.

## Graphical abstract

**Figure:**
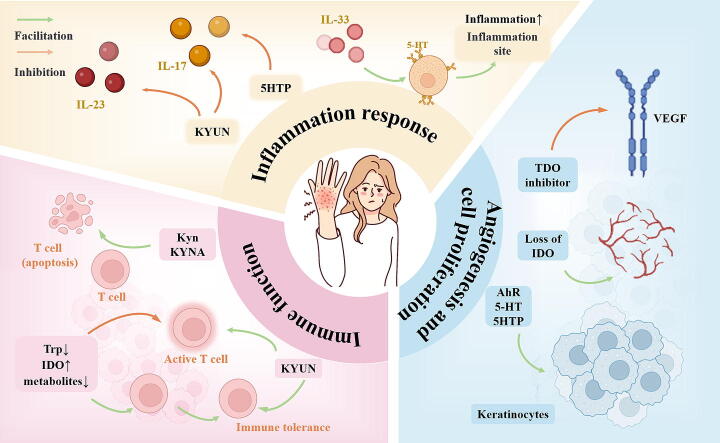

## Introduction

Psoriasis, a chronic inflammatory skin condition, impacts roughly 2–3 % of individuals worldwide. In the 2016 Global Report on Psoriasis, WHO defined psoriasis as a disease with no cure, which can occur at any age, and markedly diminishes the quality of life for affected individuals. The disease is more common in adults than children, is unevenly distributed geographically, and is more prevalent in high-income countries and areas with aging populations [1]. Although the age-standardized incidence rate for psoriasis is expected to decline by 2040, the number of affected individuals will continue to increase [2]. The pathogenesis of psoriasis involves multiple factors and remains unclear, involving genetic predisposition [3], environmental triggers, and immune dysregulation. Recently, the pivotal role of complement C3 in psoriasis has been elucidated, providing novel insights and a theoretical foundation for therapeutic targets in the treatment of psoriasis [4]. The essence of the disease is the abnormal differentiation of keratinocytes, parakeratosis, and inflammatory cell infiltration [5]. Psoriasis manifests through various clinical phenotypes, with psoriasis vulgaris being the most prevalent form. Other less frequently encountered types include guttate, erythrodermic, and pustular psoriasis [6]. Several diseases, including psoriatic arthritis, obesity, depression, cardiovascular disease, and metabolic syndrome, are closely linked to psoriasis. Psoriasis and its complications involve a variety of metabolic abnormalities, and exploring their mechanism has a significant impact on clinical treatment.

Tryptophan (Trp) is classified as an essential human amino acid with a wide range of physiological functions in regulating growth and feeding, mood and behavior, and immune responses, only be supplemented through diet. Food-borne Trp can enter the blood circulation after passing through the intestinal epithelium and reach specific sites to exert its effects. Approximately 1 % of ingested Trp is utilized for protein synthesis, the majority of Trp is metabolized through three main pathways: the kynurenine (Kyn) pathway, the serotonin (5-HT) pathway, and the indole pathway. Trp and metabolite levels are strongly correlated with some diseases, including colitis, irritable bowel syndrome depression, liver cancer, and others [7].

In recent years, emerging evidence has indicated that key enzymes and metabolites involved in Trp metabolism, such as 5-hydroxytryptamine (5-HT), kynureninase (KYNU), quinolinic acid (QA), and indoleamine-2,3-dioxygenase-1 (IDO1), are closely associated with psoriasis [8]. This paper provides a comprehensive overview of the Trp metabolic routes based on the available literature, and highlights the most relevant diseases associated with each pathway. We further review the essential enzymes and metabolites involved in Trp metabolism that contribute to the pathogenesis of psoriasis, as well as the current basic and clinical research in this area. Additionally, we discuss the complications associated with psoriasis and the involvement of Trp metabolism in its treatment strategies.

## Trp metabolism pathways

### Kyn pathway

The Kyn pathway serves as the predominant metabolic route for Trp, which is predominantly metabolized into various bioactive substances in the liver. This pathway is regulated by three rate-limiting enzymes: tryptophan-2, 3-dioxygenase (TDO), IDO1 and IDO2. TDO is almost exclusively expressed in the liver, whereas IDO is present in several human organs, including the brain, liver, and gastrointestinal system. Initially, these three rate-limiting enzymes convert Trp to N-formyl kynurenine (NFK), which is then deformylated to Kyn by arylformamidase. Subsequently, Kyn is metabolized into three distinct substances—kynurenic acid (KYNA), 3-hydroxykynurenine (3-HK), and anthranilic acid (AA)—via three different enzymes: Kyn aminotransferases (KAT I—KAT IV), KYNU, and kynurenine-3-monooxygenase (KMO) [9]. 3-HK can be converted into 3-hydroxyaminobranilate (3-HAA) and alanine by KYNU catalysis, while the transformation of 3-HK into xanthinic acid (XA) is catalyzed by KAT. Alanine undergoes transamination to produce pyruvate. 3-HAA is further metabolized by 3-hydroxy anthranilic acid 3,4-dioxygenase (3-HAO) into a compound called amino carboxy muconate semialdehyde (ACMS), which is subsequently converted into 2-aminomuconic-semialdehyde (2-AS) by ACMS decarboxylase (ACMSD). 2-AS can either be non-enzymatically cyclized to produce picolinic acid (PA) or non-enzymatically converted to QA, which is then transformed into NAD + by QA phosphor ribosyl transferase (QPRT) [10]. KYNA synthesized by astrocytes is believed to exert neuroprotective effects. In contrast, QA, produced by activated microglia, can activate the N-methyl-D-aspartate (NMDA) receptor signaling pathway, resulting in excitotoxicity and exacerbating the inflammatory response [11].

### 5-HT pathway

In the human body, a very small fraction of Trp is metabolized in the gut and brain via the 5-HT pathway. Trp is catalyzed to 5-hydroxytryptophan (5-HTP) by Trp hydroxylase (TPH). Aromatic L-amino acid decarboxylase, in conjunction with the cofactor pyridoxal-5′-phosphate, converts 5-HTP to 5-HT, a well-known monoamine neurotransmitter that controls behavior and mood, in addition to performing other critical central nervous system functions. 5-HT is subsequently transformed into 5-HIAA, which is excreted by the kidneys. The human body contains two distinct serotonin (5-HT) systems: the central and peripheral systems. In the brain, neurons primarily produce central 5-HT through TPH2. More than 90 % of the peripheral 5-HT is produced by the TPH1 enzyme in enterochromaffin cells (EC cells) and is stored in platelets. Platelet activation releases 5-HT, which binds to 5-HT receptors in the bone and liver. However, peripheral and central 5-HT systems are not interconnected, and peripheral 5-HT cannot modulate central nervous system activity or traverse the blood–brain barrier[12]. 5-HT is also catalytically converted to melatonin, which plays a major role in regulating the sleep-wake cycle. Immunocytes in the skin contain 5-HT and 5-HT biosynthetic enzymes, as well as 5-HT receptors[13,14]. Compared to normal skin, 5-HT is expressed in psoriatic lesions, and its role in inflammation may involve stimulating keratinocyte proliferation and directly affecting CD4 + T[15]. Additionally, the 5-HT2AR, which is known to participate in pro-inflammatory effects, is highly expressed in the dermis of psoriasis, contributing to the recruitment of CD4 + T to inflammatory sites, thereby exacerbating the inflammatory response[16].

### Indole pathway

**Fig. 1:**
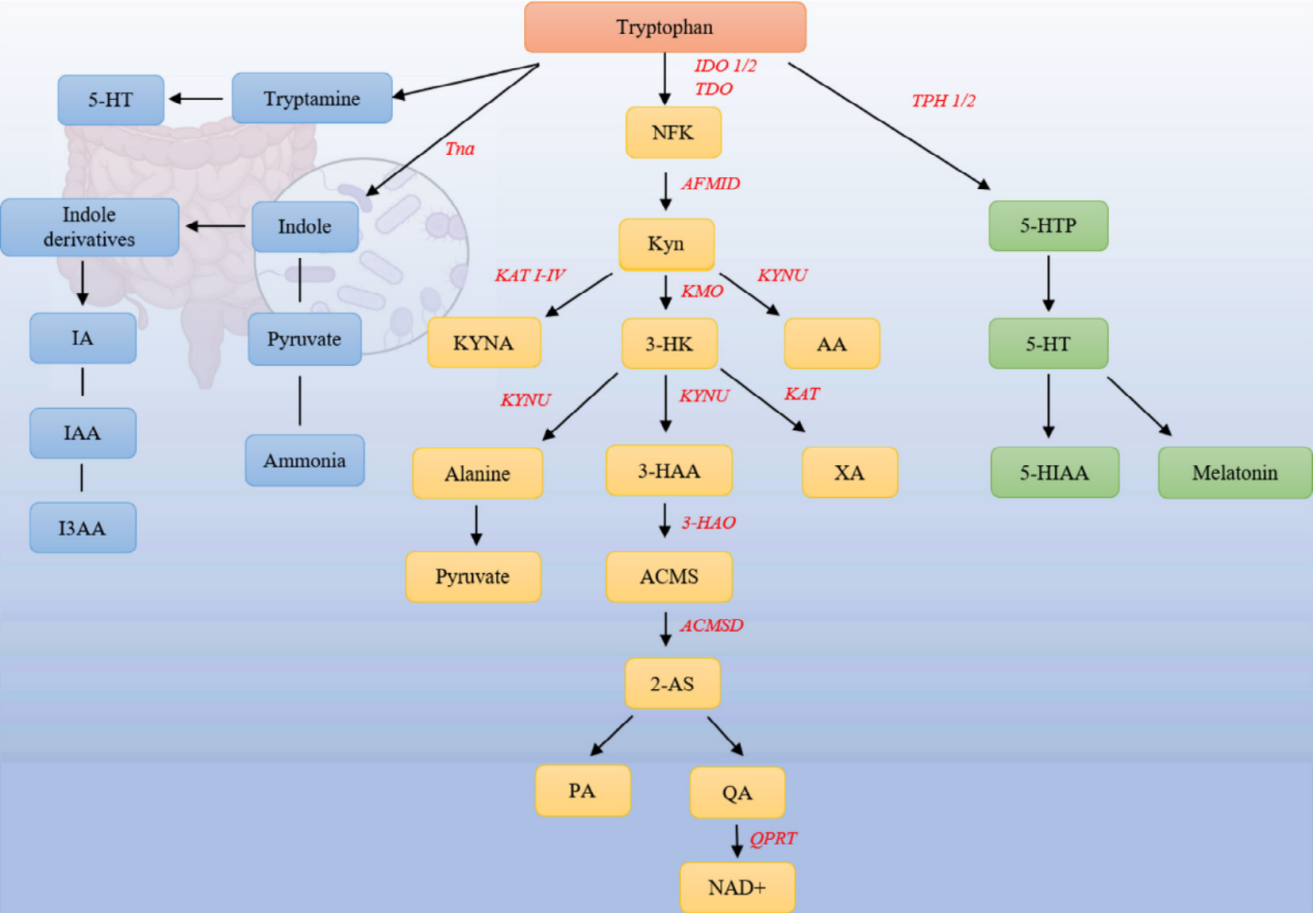
Tryptophan (Trp) catabolic pathway. Trp is catabolized through three pathways, namely the serotonin (5-HT) pathway (right), the indole pathway (left), and the Kynurenine (Kyn)pathway (middle).

A minor fraction of Trp is metabolized into indole and its derivatives by gut microbiota. The microorganisms involved in this process include *Clostridium spores*, *Clostridium botulinum*, *Lactobacillus*, *Peptostreptococcus* spp. Indole and its derivatives include indole acrylic acid (IA), indole-3-acetic acid (I3AA), indole-3-acetaldehyde (3-IAld), and indole-3-propionic acid (IPA). Tryptophanase (Tna) serves as the primary enzyme responsible for catalyzing the conversion of tryptophan (Trp) into indole, pyruvate, and ammonia, utilizing pyridoxal 5′-phosphate as a cofactor, and it is also essential for converting Trp to indole in *Escherichia coli* [17]. Trp can also be converted into other substances through alternative pathways. For example, Trp can be decarboxylated by *C. sporogenes* and *Ruminococcus gnavus* to produce the neurotransmitter tryptamine, which promotes 5-HT secretion by EC cells and subsequently stimulates gastrointestinal motility by affecting enteric neurons. IAA can be converted into 3-methylindole by *Bacteroides* spp. and *Clostridium* spp in a similar manner [18,19]. Additionally, microorganisms that influence Kyn metabolism, such as *Pseudomonas*, *Staphylococcus*, and *Providencia*, can accelerate the conversion of Trp to Kyn. It has been speculated that the phenylacetate dehydratase gene cluster in *Peptostreptococcus* spp contributes to the conversion of Trp to IA and I3AA[20]. (Fig. 1).

### Dysregulated tryptophan metabolism in psoriasis

Early studies on IDO have shown that this enzyme has immunomodulatory effects in infection, autoimmune processes, tumor growth, and organ transplantation. In the pathophysiology of psoriasis, the dysregulation of Trp metabolism plays a crucial and multifaceted role, particularly the Kyn pathway. Compared with healthy controls, patients with psoriasis have higher levels of serum IDO and Kyn. A significant stimulation of the Kyn pathway in serum patients with psoriasis suggests its role in its pathogenesis[21]. However, as the severity of psoriasis increases, IDO activity significantly decreases. This reduction is attributed to the inability of peripheral immune cells from psoriasis patients to effectively upregulate IDO expression in response to inflammatory stimuli. This defect not only impairs the differentiation of regulatory T cells (Tregs) but may also exacerbate the inflammatory response characteristic of psoriasis. Studies have shown that this defect in IDO expression correlates negatively with disease severity, suggesting that decreased IDO activity may be a significant marker of worsening psoriasis[22]. All the above studies provide substantial evidence to clarify the relationship between psoriasis and IDO, and also reflect the complexity of the relationship between them. Given the role of IDO in tumor immune evasion through the promotion of Trp metabolism, a large number of studies have focused on IDO inhibitors. Numerous researchers have demonstrated a strong association between IDO and Th17/Treg balance that drives psoriatic pathogenesis[[23], [24], [25]], it is important to explore the relationship between psoriasis and IDO. After all, psoriasis is a chronic, intractable condition that causes great suffering and stigma among patients.

As can be seen from Fig. 1, there is an enzyme named KYUN with a high metabolic involvement downstream of Kyn. It exerts pro-inflammatory effects, thereby contributing to the pathogenesis of psoriasis[26]. The KYNU expression is significantly upregulated in psoriatic lesions and correlates positively with disease severity and inflammation levels. Successful treatment of psoriasis is associated with a reduction in KYNU expression. Cells expressing KYNU (referred to as KYNU + ) are predominantly of myeloid origin, and their infiltration is augmented in psoriatic lesions. Trp metabolites downstream of KYNU, such as 3-HK and QA, promote the production of diverse inflammatory mediators, including cytokines, chemokines, and adhesion molecules. Conversely, metabolites upstream of KYNU, such as KYNU itself, do not elicit this effect and may even attenuate the expression of certain inflammatory factors. In summary, the collective evidence suggests that KYNU could potentially function as a biomarker for psoriasis [27,28]. Another Kyn downstream metabolite, KYNA, has an inhibitory effect on the frequency of IL-23-producing DCs, and also the level of IL-17 mRNA and the frequency of IL-17 + T cells [29]. This is particularly significant for psoriasis, which is driven by the IL-23/IL-17 axis.

Angiogenesis is one of the pathological features of psoriasis and also one of its pathogeneses. Anti-VEGF therapy in mice with psoriasis symptoms exhibited complete improvement in skin lesions and a significant reduction in blood vessels [30]. TDO emerges as a pivotal player in the proliferation of human umbilical vein endothelial cells (HUVECs) and human endothelial colony-forming cells (ECFCs), as well as in capillary morphogenesis in vitro, suggesting its potential pathophysiological role in angiogenesis beyond its well-known immunomodulatory effects [31]. In mouse models that are deficient in IDO, neovascularization was significantly reduced [32]. This finding may have important implications for the development of IDO inhibitors, particularly in the treatment of psoriasis associated with neovascularization.

A clinical study utilizing immunohistochemical techniques suggests that 5-HT may contribute to the development of psoriasis through its dual function: facilitating keratinocyte proliferation, mediating inflammatory responses, and stimulating T-cell activation[33]. Specifically, 5-HT significantly stimulates keratinocyte proliferation [34] and enhances cell migration and G1 phase progression in HaCaT cells via the 5-HT2BR/ERK pathway [35]. Interleukin-33 (IL-33), a potent activator of mast cells (MCs), is secreted by keratinocytes in psoriasis. MCs are activated by IL-33 during the early stages of the disease, thereby exacerbating psoriasis-associated skin inflammation [36]. Additionally, 5-HT can exacerbate inflammation by binding to the 5-HT1A receptor (5-HT1AR) on these cells, thereby recruiting them to the inflammatory site [37]. Pain is one of the chief complaints among patients with psoriasis. A study using an animal model has shown that subcutaneous injection of 5-HT receptor agonists can significantly reduce long-term allodynia and hyperalgesia induced by IMQ [38]. Current clinical treatments for pain in psoriasis include biological agents and traditional oral drugs, each with its advantages and disadvantages. Based on the findings of this study, 5-HT receptor agonist treatment may be a viable option when depression and pain coexist in patients with psoriasis. Meanwhile, 5-HTP alleviates IMQ-induced psoriasiform dermatitis, likely by inhibiting IL-17A production and keratinocyte activation [39]. Additionally, melatonin and its metabolite promote differentiation in the human epidermis and contribute to the maintenance of the skin barrier [40]. Overall, the metabolites discussed above hold potential as biomarkers or therapeutics for psoriasis.

Studies have shown decreased intestinal microbiome diversity in psoriasis patients compared to controls and decreased beneficial microbial species, including *Parabacteroides*, *Coprobacillus*, and *Faecalibacterium prausnitzi* [41], and excessive abundance of harmful bacteria, including *Escherichia coli*, *Campylobacter*, *Helicobacter*, *Salmonella*, *Alicaligenes*, and *Mycobacterium* species has been observed [42]. The influence of tryptophan and its metabolites on intestinal bacteria is primarily mediated through the regulation of immune function or the modulation of immune cells. For instance, I3AA has been shown to inhibit the infection of *Helicobacter pylori* while promoting the growth of *Coprobacillus* bacteria. Similarly, IPA enhances the proliferation of *Parabacteroides*, and I3AA supports the growth of *Faecalibacterium prausnitzii*. These actions collectively contribute to the improvement of gut microbiota structure in psoriasis [43].

The aryl hydrocarbon receptor (AhR) is a ligand-activated transcription factor that plays a pivotal role in modulating inflammation and immune cell differentiation, particularly in the context of psoriasis [44]. Activation of AhR has been shown to promote the differentiation of Tregs and influence the expansion of Th17 cells, thereby maintaining immune homeostasis. Kyn, a key metabolite in the tryptophan catabolism pathway, serves as an endogenous agonist of AhR, upregulating the expression of immunosuppressive genes such as TGFB1 and IDO in a dose-dependent manner. Additionally, indole derivatives, which are also AhR agonists, have been shown to enhance the expression of antimicrobial peptides in the gut, thereby maintaining microbiota homeostasis. These mechanisms collectively contribute to the beneficial effects observed in psoriasis. Recently, the AhR agonist, Tapinarof cream has been approved for the treatment of psoriasis, marking a significant breakthrough in the development of novel therapeutic strategies for this chronic skin condition [45].

**Fig. 2:**
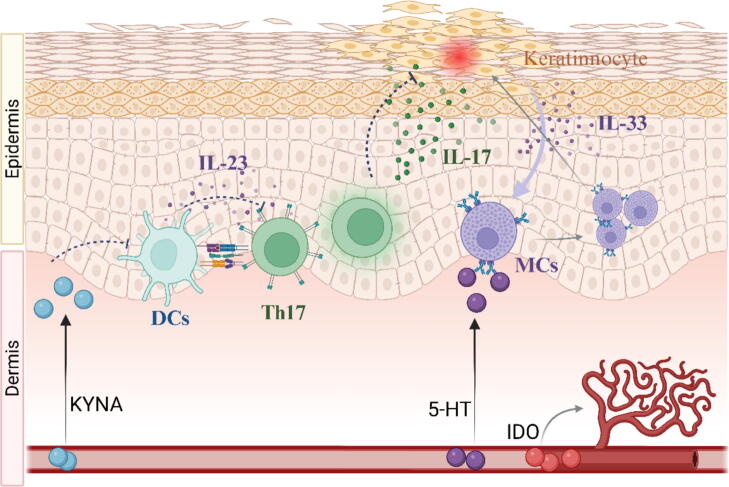
Kynurenic acid (KYNA) diminishes reduces the frequency of IL-23 production by dendritic cells (DCs) and IL-17 levels in Th17 cells, thereby curbing keratinocyte proliferation and inflammation. Early-stage keratinocytes secrete IL-33, a potent mast cell activator. Moreover, serotonin (5-HT) can bind to surface receptors on mast cells (MCs), recruiting them to inflammatory sites and exacerbating the inflammatory response. Indoleamine 2,3-dioxygenase (IDO) expressed in vascular endothelial cells promotes capillary morphogenesis and contributes to psoriasis pathogenesis.

From a practical standpoint, IDO has been prioritized as a therapeutic target over KYNU, given the extensive body of evidence from cellular, animal, and human studies, as well as the clinical use of IDO inhibitors. These studies have provided substantial support for the role of IDO in various inflammatory and autoimmune conditions, including psoriasis. However, recent studies have unveiled a more definitive connection between the downstream metabolites of Kyn and psoriasis, highlighting the beneficial effects of KYNA and other AhR ligands on the disease. These findings suggest that therapeutic strategies targeting KYNU may hold greater promise than those targeting IDO, warranting further investigation. Nevertheless, the specific choice of medication in actual treatment should still be determined based on the individual patient's condition, taking into account factors such as disease severity, comorbidities, and potential side effects. Future research should continue to explore the complex interplay between AhR, tryptophan metabolites, and psoriasis, with the aim of developing more effective and targeted therapeutic interventions. (Fig. 2).

### Abnormal Trp metabolism in psoriasis and its comorbidities

#### Trp metabolism abnormalities in psoriasis vulgaris

While psoriasis can present in a variety of clinical forms, persistent plaque psoriasis, commonly referred to as psoriatic vulgaris (PV), is the most prevalent and easily identifiable type. Its primary clinical signs include silvery scales and prominent erythema. Any area of the skin may be affected, although the elbow, waist, and scalp are common sites. Topical therapy is the most widely used treatment for PV. For mild psoriasis, topical therapy is the most effective treatment; however, systemic therapy and ultraviolet radiation are recommended for individuals presenting with moderate-to-severe psoriasis. Because of tissue-resident memory T-cells, psoriasis is challenging to treat and prone to recurrent attacks [46]. A 2025 case-control study (n = 60) reported significantly higher serum and urine Kyn, KYNA and QA in PV patients than in matched controls, indicating chronic overstimulation of the Kyn pathway. IDO activity correlated positively with PASI and with circulating IL-17A, supporting Kyn pathway activation as a marker and amplifier of cutaneous inflammation [8]. Although these data are cross-sectional, they provide a rationale for Kyn pathway −directed biomarkers and for testing topical AhR modulators (already approved for PV) as Kyn pathway fine-tuners. A study exploring the role of KYUN in psoriasis revealed that it is the downstream products of KYUN, rather than KYUN itself, that have pro-inflammatory effects. Its levels increase in parallel with the severity of the disease and inflammation, and it decreases after successful treatment of psoriasis [28]. This provides an important basis for targeted Trp metabolism therapy for psoriasis.

#### Trp metabolism abnormalities in guttate psoriasis

Guttate psoriasis (GP), a form of psoriasis, is typically triggered by streptococcal infection, such as pharyngitis or perianal infections, and predominantly affects children and adolescents rather than adults. GP, which accounts for 2 % of all psoriasis cases, is characterized by a centripetal pattern of numerous small, scaly papules. Approximately 40 % of children and young people with GP develop chronic plaque disease, although the majority experience spontaneous regression after weeks or months. T cells assist in the pathogenesis of psoriasis, with evidence supporting the correlation between GP and T cell superantigen stimulation triggered by streptococcal superantigens [47]. A study investigated whether exposure of human peripheral monocytes to streptococcal-derived superantigens is linked to Trp degradation in vitro. The findings revealed that streptococcal erythrogenic toxins induce IFN-γ production in peripheral blood mononuclear cell cultures, and this cytokine induces IDO, which degrades Trp to form Kyn [48]. This leads to a transient surge in Kyn/Trp ratio, which may amplify Th1/Th17 responses during acute flares. Longitudinal trials are warranted to verify whether short-course oral IDO inhibitors prevent GP conversion, it could theoretically blunt the Kyn/Trp surge associated with guttate flares.

#### Trp metabolism abnormalities in erythrodermic psoriasis

Erythrodermic psoriasis (EP) is a rare variant of psoriasis, often a complication of a psoriatic flare, but potentially life-threatening, accounting for approximately 1 % of cases. During the initial phase of the disease, redness appears in the area of the original psoriasis lesion, rapidly spreads to large areas, and eventually involves the entire body with extensive redness. Patients with EP usually require hospitalization and can be treated with gentle medicated baths in addition to fluid infusion and anti-infective therapy. The commonly used drugs for the treatment of EP include retinoids, which can be taken orally, and immunosuppressants such as cyclosporine A and methotrexate. A recent clinical study performed metabolomics analysis in patients with EP and found that the differential metabolites were enriched in amino metabolism and glycerophospholipid metabolism. The levels of Trp in EP patients’ serum were lower and negatively correlated with the Psoriasis Area and Severity Index (PASI) score [49]. Dysregulated amino acid and glycerophospholipid metabolism suggests a systemic catabolic state, possibly driven by prolonged IDO activation and excessive Trp consumption. The Trp depletion may impair Treg function and exacerbate systemic inflammation. EP is associated with systemic Trp depletion, likely reflecting chronic immune activation. Trp levels may serve as a metabolic indicator of disease severity.

#### Trp metabolism abnormalities in pustular psoriasis

Pustular psoriasis is a rare disease with unique morphology, characterized by sterile pustules and erythema. Based on the anatomical site of involvement, it is divided into three subgroups: generalized pustular psoriasis (GPP), palmoplantar pustular psoriasis (PPP), and acrodermatitis continua of Hallopeau (ACH) [50]. GPP and PPP are the most common subtypes. The onset of GPP is typically acute and can be triggered by infection or drug use. Clinically, it manifests as the appearance of needle-to-miliary-sized, superficial sterile pustules covered with atypical white scales on a background of psoriatic lesions. PPP predominantly occurs on the feet, with skin lesions on the palms and soles. The rash is characterized by deep-seated sterile pustules on a background of erythema, often with thick, adherent scales. Pustular psoriasis cannot currently be cured and is prone to recurrent attacks, especially PPP, which significantly impacts patient's daily lives due to its localization on the palms and soles. GPP is characterized by acute, sterile pustule formation and innate immune dysregulation. Metabolomics and functional studies of GPP serum and monocytes have revealed that the depletion of serum amino acids, including Trp, attenuates the innate immune response of blood monocytes through the amino acid response pathway. This Trp depletion activates the amino acid response (AAR) pathway, which acts as a negative feedback mechanism to restrain monocyte hyperinflammation [51]. Thus, Trp metabolism in GPP may serve a dual role: fueling inflammation via Kyn pathway and simultaneously activating counter-regulatory pathways. Focusing on levels of the amino acid in the bloodstream helps monitor disease severity, shed light on the pathogenesis of GPP and, facilitate the development of therapeutic strategies.

#### Trp metabolism abnormalities in psoriatic arthritis

A psoriatic rash is often accompanied by pain, swelling, soreness, stiffness, and dyskinesia in the joints and surrounding soft tissues. The disease burden is high and can occur at any age, with no significant gender differences, although spinal involvement is more common in males. Treatment aims to relieve pain, delay joint destruction, and control skin damage. Treatment options vary from person to person. Epidemiological findings show an estimated 17.58 % for PsA in patients with psoriasis[52]. Early detection of Trp and inflammatory factors in PsA synovial fluid revealed that Trp levels are associated with the proportion of polymorphonuclear cells and directly correlated with IL-1β[53]. The lining layer of the synovium is composed of fibroblast-like synoviocytes and resident macrophages, which are essential to the development and actively promote joint degradation[54]. Additionally, PsA causes the synovium to overexpress cadherin 11, a crucial protein involved in the cytoskeletal dynamics of fibroblast-like synoviocytes[55]. Reports indicate that higher expression of TDO2 can result in synovial inflammation and joint destruction, accelerating the process of disease[56]. TDO2-mediated Trp degradation in the synovium contributes to joint inflammation in PsA, Therefore, targeting the Kyn pathway of TDO2 activity and Trp degradation may represent potential therapeutic strategies.

#### Trp metabolism abnormalities in depression

Depression is a mental disorder characterized primarily by a significant and enduring low mood, some patients may experience self-injury and suicide, and may even have delusions, hallucinations, and psychotic symptoms. Individuals with psoriasis experience significant stigmatization due to the chronic, recurrent, and highly visible nature of their skin lesions. This stigmatization has profound negative impacts on their social life, interpersonal relationships, and overall quality of life, often leading to the development of psychological issues. Recent studies have highlighted that within this affected population, the prevalence of depression, anxiety, and suicidal ideation has reached 20 %, 21 %, and 0.77 %, respectively [57]. Psoriasis can cause or aggravate the symptoms of depression, and depression also can exacerbate psoriasis, causing a malignant cycle between psoriatic and depression. There is a large number of clinical evidence that patients with psoriasis have depression, and there are also theories that clarify the correlation between the two, the theory of inflammation, the gut-brain-skin axis, has laid the theoretical foundation for finding novel therapeutic approaches to psoriasis combined with depression [58]. IMQ-induced psoriatic mice showed depressive-like behavior, which was manifested as lower sucrose preference, decreased desire to explore new things, and decreased hypothalamic 5-HT level. Patients with psoriasis and comorbid depression exhibit lower hippocampal 5-HT levels and higher serum Kyn/Trp ratios, indicating shifted Trp metabolism toward neurotoxic the Kyn pathway metabolites at the expense of 5-HT synthesis. This may contribute to mood deterioration and increased pain perception. 5-HT was significantly correlated with PASI in patients with symptoms of depression, but was not associated with PASI in those without depression [59]. A population-based cohort study showed that the use of antidepressant selective 5-HT transporter inhibitors (SSRIs) can reduce the systemic use of medication in patients with psoriasis [60]. Another study showed that major depression is an independent risk for psoriasis, while long-term treatment with SSRIs can reduce the risk of major depression complicated with psoriasis [61,62], indicating that SSRIs have a protective effect on psoriasis. In summary, restoring 5-HT balance via SSRIs may offer dual benefits for mood and inflammation.

#### Trp metabolism abnormalities in metabolic syndrome

Metabolic syndrome (MetS) refers to the pathological state characterized by metabolic disorders of protein, fat, carbohydrate, and other substances in the human body. It is defined by the coexistence of multiple metabolic abnormalities and increases the risk of cardiovascular disease. In individuals with psoriasis, the prevalence of metabolic syndrome (MetS) varies between 20 % and 50 %, escalating with the increasing severity of psoriasis [63]. Although a definitive cause-and-effect relationship between psoriasis and MetS has not been established, psoriasis patients may exhibit metabolic abnormalities due to a combination of environmental factors, shared signaling pathways, and genetic predispositions [64]. The primary clinical treatments include adjusting dietary adjustments and physical exercise to promote weight loss, as well as pharmacological interventions to reduce insulin resistance and improve lipid profiles. A recent report results showed an inverse association between dietary Trp intake and MetS incidence, even after adjustment for multiple confounding factors, such as nutrients and food patterns [65]. Urinary analysis in MetS patients showed markedly elevated levels of Kyn, KYNA, and QA, along with an increased Kyn-to-Trp ratio [66]. Urinary Trp metabolite analysis may serve as a means to identify biomarker for MetS. Collectively, Trp metabolism is dysregulated in MetS, with elevated Kyn pathway metabolites potentially driving metabolic dysfunction. Modulating Kyn pathway activity may mitigate cardiovascular comorbidities in psoriasis patients and reduce cardiovascular risk.

#### Trp metabolism abnormalities in inflammatory bowel disease

IBD, encompassing Crohn's disease and ulcerative colitis, is defined by recurrent and distinct clinical presentations. It is characterized by chronic inflammation in various regions of the gastrointestinal tract, which can cause diarrhea and abdominal pain. Several studies have confirmed the correlation between psoriasis and IBD, with mechanisms potentially involving changes in gut microbiota, genetic deletion of clusterin, and TLR7-dependent eosinophil degranulation [[67], [68], [69]]. A clinical study observed an inverse correlation between serum Trp levels and disease activity. Elevated levels of Trp metabolites, particularly QA, indicate high Trp degrading activity in patients with active IBD patients. Trp deficiency and Kyn pathway activation may promote the development of IBD or exacerbate disease activity [70]. Therefore, researchers speculate that reduced Trp levels, due to malabsorption or colonic leakage, may affect IL-22 secretion and thus maintain an inflammatory state [71]. AhR is known to induce the production of IL-22, which plays a positive role in the regeneration and protection of the intestinal mucosa [72]. IBD shows reduced levels of tryptophan indole pathway metabolites IPA and IAA, which can activate AhR and promote AhR's beneficial effects on the gut. Therefore, promoting Trp intake and indole pathway metabolism, while inhibiting the Kyn pathway, can improve intestinal symptoms in IBD.

Across all reviewed comorbidities, Trp metabolism is consistently shifted toward the kynurenine pathway, resulting in Trp depletion, neurotransmitter imbalance, and immune dysregulation. Key enzymes such as IDO1, KYNU, and TDO2 emerge as central regulators, while metabolites like Kyn, and QA serve as both biomarkers and effectors of disease activity. Targeting Trp metabolism—via enzyme inhibitors, AhR modulators, or dietary interventions—holds promise as a transversal therapeutic strategy for psoriasis and its systemic complications. (Table 1).

**Table 1:** Changes in Trp metabolism identified in studies of psoriasis and its complications.

Type of Psoriasis	Characteristics of the Study	Samples	Changes in Trp catabolism	Specific value	References
PV	Patients with PV	Skin	↓IDO	Normal VS nonlesional psoriatic skin * Nonlesional psoriatic skin VS lesional psoriatic skin **	[28]
			↓KYNU	Normal VS lesional psoriatic skin ** Nonlesional psoriatic skin VS lesional psoriatic skin ***	
GP	Human	PBMC	↓Trp	Con VS ET *	[48]
EP	20 patients with EP	Serum	↓Trp	Con VS EP ***	[49]
GPP	24 GPP patients	Serum	↓Trp	Con VS PV * Con VS GPP * PV VS GPP ***	[51]
PsA	13 patients with PsA	SF	↓SF Trp	PsA VS osteoarthritis **	[73]
Depression	PV mice	hippocampus	↓5-HT	Con VS model ***	[74]
	113 patients with PV	Serum	5-HT was significantly correlated with PASI in patients with symptoms of depression	r = 0.50	[59]
MetS	182 patients with metabolic syndrome	Urine	↓3-HK	ConVS MetS ***	[75]
			↓QA	Con VS MetS *	
			↓Kyn	Con VS MetS ***	
			↓XA	Con VS MetS ***	
IBD	Patients with IBD	Serum	↓Trp	Con VS IBD ***	[70]
		Colon	↓TDO	Con VS IBD ***/*	
			↑IDO	Con VS IBD ***/**/*	

### Significance of tryptophan metabolism in psoriasis therapy

#### Medications

It has been discovered that several clinically utilized medications have an impact on Trp catabolism while treating psoriasis. Biological agents are currently a very popular treatment method because they can directly target the inflammatory factors that cause skin lesions. Meanwhile, biological agents regulate the metabolism of tryptophan. Studies have shown that KYNU expression was significantly decreased in microarray analysis of patients treated for 12 weeks with biological therapies, including etanercept (anti-TNFα) and usekizumab (anti-IL-12/23p40). Reduced the metabolites downstream of KYUN that promote the expression of inflammatory factor genes [28]. Formylindolo[3,2-b] carbazole (FICZ), a Trp photoproduct generated by UVB exposure, can mitigate the harmful effects of UVB exposure. This is because FICZ has biological effects similar to those of UVB, such as AhR activation and ROS generation [76]. Tapinarof 1 % cream, the first small-molecule topical AhR modulator, has received approval for treating adult plaque psoriasis [45]. It introduces a mechanistically distinct option: by antagonising endogenous AhR ligands generated through the tryptophan–kynurenine pathway (e.g., kynurenine and FICZ), it re-balances T-helper-cell differentiation, down-regulates IL-23/IL-17 signalling, enhances immunosuppressive circuits and restores epidermal barrier proteins. Furthermore, triazolopyridine derivatives, a novel family of AhR agonists, exhibit significant anti-psoriatic activity and favorable pharmacokinetic properties, making them a promising candidate for psoriasis treatment [77]. The use of AhR modulators not only mitigates the carcinogenic risks associated with ultraviolet therapy, but also addresses the issue of insufficient efficacy when drugs are administered locally. Even though current clinical studies and data indicate that the modulator is generally safe under appropriate dosages and usage conditions, the diverse functions of AhR suggest potential safety risks in clinical use. Therefore, it is necessary to monitor adverse reactions and conduct long-term follow-up studies to assess their safety during clinical application. Ongoing research is crucial for a better understanding of the mechanisms of action and potential risks associated with AhR modulators, and it also aids in the development of more effective monitoring and evaluation strategies. Fumarate esters are oral systemic therapeutic drugs for treating psoriasis. Dimethyl fumarate (DMF) and its active metabolite, monomethyl fumarate (MMF), are generally regarded as the pharmacologically active components of it. Studies have shown that DMF and MMF can significantly inhibit the IDO activity induced by IFN-γ in a NF-κB signaling pathway-dependent manner within therapeutic concentrations, reduce the conversion of tryptophan to kynurenine, and also inhibit the expression of KYUN [78].

#### Dietary intervention

Psoriasis requires lifelong management. However, controlling it solely with medication is not suitable for many patients, especially during remission or recovery periods. Non-drug alternative therapies can effectively alleviate patients’ symptoms. The Mediterranean diet (MD), characterized by a high intake of nuts, fruits, vegetables, grains, legumes, fish, and olive oil and a low intake of saturated fat, is a healthy and sustainable diet. The MD is rich in antioxidants, trace elements, minerals, and vitamins and features anti-inflammatory properties. It can also exert anti-inflammatory effects by enhancing autophagy and correcting Th cell imbalances, regulating cell adhesion molecule expression, influencing DNA methylation, Modulating the gut microbiome and immune system [79]. The MD diet can enhance gut homeostasis by regulating the gut microbiota, which in turn affects Trp catabolism. The compliance of patients with the Mediterranean diet and their disease scores are negatively correlated [80], indicating that the MD has a beneficial effect on improving the severity of the disease. The MD may have positive effects on the disease management and treatment of psoriasis in adults and children.

Patients with psoriasis have a higher susceptibility to intestinal homeostasis imbalance compared to healthy individuals [81]. Increasing the intake of probiotics is also an important measure for controlling the progression of the disease. According to current research, probiotics can restore intestinal homeostasis by regulating the composition of intestinal microorganisms, restoring the intestinal barrier, inhibiting the IL-23/Th17 axis, increasing the level of Trp-AhR ligands and increasing the production of SCFAs, thereby improving the inflammatory state and alleviating the severity of the disease [82,83]. The results of a randomized clinical trial involving the use of a probiotic mixture indicate that a possible positive effect of the probiotic mixture in reducing the severity of psoriasis when administered to patients with psoriasis together with the topical corticosteroid betamethasone and calcipotriol [84]. It has been confirmed that Bifidobacterium longum is a key catalyst for catalyzing the synthesis of indole derivatives from Trp, and it can also improve the side effects of medications without affecting their efficacy. B longum treatment maintained barrier integrity by increasing the abundance of propionate in the gut, thereby regulating the balance of Th17/Treg cells [85]. Probiotics has a higher rate of patient compliance and are more convenient to use, which may lead to significant therapeutic benefits in the long term. Prebiotics, which act as substrates for probiotics, selectively enhance the proliferation and metabolic activity of beneficial intestinal microbiota. In a clinical trial, the oral administration of a probiotic consortium comprising five Bacillus spore strains over a 12-week period in conjunction with a prebiotic regimen consisting of fructo-oligosaccharides, *xylo*-oligosaccharides, and galacto-oligosaccharides over an 8-week period significantly enhanced the therapeutic efficacy among patients with psoriasis receiving topical treatment [86].

#### Exercise

**Fig. 3:**
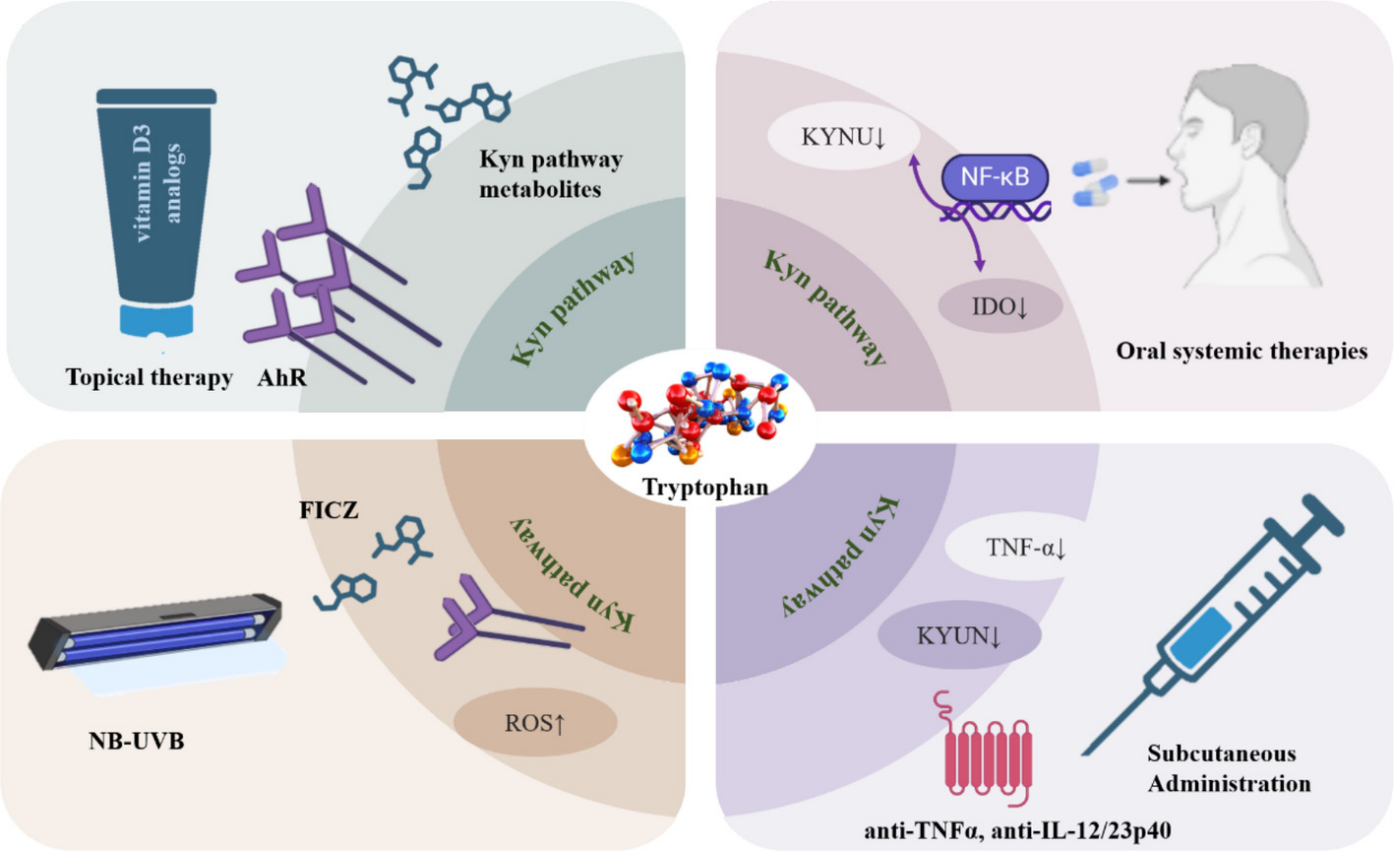
Common treatment modalities for psoriasis and associated tryptophan metabolism.

Having psoriasis can cause embarrassment and depression, which can prevent people from wanting to exercise. Studies have shown that patients with psoriasis who engage in less exercise have more severe symptoms and lower scores on the Dermatology Life Quality Index (DLQI). Especially for women, it seems that they tend to avoid physical exercise. Exercise improves mood by increasing the utilization of tryptophan, enhancing serotonin synthesis and regulating the kynurenine pathway. Physical exercise enhances the synthesis of KYNA by activating KATs [87]. KYNA has neuroprotective and anti-inflammatory effects and may have positive impacts on diseases such as psoriasis. Obesity is a significant risk factor for psoriasis. Dysregulation of skin-resident PPARγ-positive Tregs induced by obesity fosters IL-17A-mediated inflammatory responses in psoriasiss [88]. A high-fat diet significantly and dose-dependent exacerbates IMQ-induced skin inflammation [89]. For obese psoriasis patients, weight loss is a crucial component of treatment. The results of a randomized trial investigating the long-term impact of weight loss on the severity of psoriasis show that during weight loss maintenance diet, PASI score decreased and DLQI increased [90]. Dietetic intervention associated with increased physical exercise reduced psoriasis severity in systemically treated overweight or obese patients with active psoriasis [91]. Moreover, weight loss can also reduce the cardiovascular risks for obese patients with psoriasis [92] (Fig. 3, Table 2).

**Table 2:** The treatment of psoriasis and its complications alters the catabolism of Trp.

Types of interventions	Intervention methods	Intervention results	Specific value	References
Topical therapy	Corticosteroid	Activated TPH1 and TPH2	Beclomethasone dipropionate VS control ***	[93]
	AhR modulator	Modulate AhR signaling	Con VS model ***	[77]
		↓ Oxidative stress by AhR	Con VS model ***	
Phototherapy	UVB	↑Viability of HEKn keratinocytes exposed to UVB	Con VS treat ***/**/*	[94]
		↓ UVB-induced ROS generation	Con VS treat ***	
		↓ Levels of NO-produced by UVB irradiated keratinocytes	Con VS treat ***	
		↑ Repair of DNA damage induced by UVB	Con VS Mel **, Con VS 6-OHM *, Con VS 5-MT *, Con VS NAS **	
		↑ GSH in UVB-exposed keratinocytes	Con VS treat *	
		FICZ induces ROS production in AhR dose-dependent manner	Con VS FICZ **/***	[76]
		FICZ induces the expression of IL-1A, IL-1B, IL-6 in a ROS-dependent manner	Con VS treat ***/**/*	
Oral systemic therapies		↓ Kyn	Con VS treat ***	[78]
Biologics	SSRIs	Decreased need for systemic psoriasis treatment	95 % confidence interval 0.28–0.68	[60]
	5-HTR agonist	↓ 5-HT in serum	Con VS model *	[38]
		↑ 5-HT in serum	Model VS treat *	
		↑ ICAM-1, VCAM-1, IL-6	Con VS model *	[95]
		↓ICAM-1, VCAM-1, IL-6	Model VS treat *	
	VEGF inhibitor	↓ The ear thickness and epidermal thickness	Model VS treat **	[30]
		↓ Size and number of vessels in the skin	Model VS treat ***	

## Conclusion and prospect

The progression of psoriasis from localized to systemic inflammation exerts diverse impacts on various tissues and organs, potentially leading to or exacerbating a range of comorbidities. These processes are significantly influenced by tryptophan (Trp) metabolites and their associated enzymes, which may serve as valuable biomarkers for clinical diagnosis and therapeutic intervention. It is anticipated that the development of novel treatments targeting the Trp metabolome and associated enzymes will alleviate or treat psoriasis and its associated complications. For instance, inhibitors of IDO, TDO, KMO, and tryptophan hydroxylase (TPH) may significantly enhance the efficacy of conventional therapies. However, further research is required to optimize dosing regimens and co-administration strategies. Although our study suggests targeting Trp metabolic enzymes for therapeutic intervention, we acknowledge the challenges in drug delivery, such as achieving tissue-specific inhibition of KMO. To enhance the translational relevance of our findings, it is imperative that we develop more precise drug delivery systems or explore more selective compounds. These endeavors are crucial for ensuring the safe and effective clinical application of our discoveries. Moreover, direct supplementation with indoles and their derivatives represents a potential therapeutic approach.

Given the crucial role of Trp metabolites in psoriasis and its complications, a comprehensive investigation of their functions and regulatory mechanisms, as well as the associated enzymes, is warranted. Utilizing more readily available clinical samples to elucidate these mechanisms will better support clinical practice and contribute to the development of targeted therapies. Future research should focus on elucidating the complex crosstalk between Trp metabolites and immune responses in psoriasis, as well as exploring the potential of Trp-based interventions in clinical settings.

## Declaration of competing interest

The authors declare that they have no known competing financial interests or personal relationships that could have appeared to influence the work reported in this paper.

## References

[1] Parisi R., Iskandar I.Y.K., Kontopantelis E., Augustin M., Griffiths C.E.M., Ashcroft D.M. (2020). National, regional, and worldwide epidemiology of psoriasis: systematic analysis and modelling study. BMJ (Clin Res Ed,).

[2] Li D.-P., Han Y.-X., He Y.-S., Wen Y., Liu Y.-C., Fu Z.-Y. (2023). A global assessment of incidence trends of autoimmune diseases from 1990 to 2019 and predicted changes to 2040. Autoimmun Rev.

[3] Babaie F., Omraninava M., Gorabi A.M., Khosrojerdi A., Aslani S., Yazdchi A. (2022). Etiopathogenesis of psoriasis from genetic perspective: an updated review. Curr Genomics.

[4] Cao Q., Li J., Zhang K. (2025). The role of complement component 3 (C3) in psoriasis. Curr. Mol Pharmacol.

[5] Guo J., Zhang H., Lin W., Lu L., Su J., Chen X. (2023). Signaling pathways and targeted therapies for psoriasis. Signal Transduction Targeted Ther.

[6] Boehncke W.-H., Schön M.P. (2015). Psoriasis. Lancet.

[7] Xue C., Li G., Zheng Q., Gu X., Shi Q., Su Y. (2023). Tryptophan metabolism in health and disease. Cell Metab.

[8] Stepaniuk A., Baran A., Hermanowicz J.M., Sieklucka B., Pawlak D., Flisiak I. (2025). Peripheral kynurenine pathway metabolites in patients with psoriasis. Int J Mol Sci.

[9] Barone P. (2019). The “yin” and the “yang” of the kynurenine pathway: excitotoxicity and neuroprotection imbalance in stress-induced disorders. Behav Pharmacol.

[10] Mor A., Tankiewicz-Kwedlo A., Ciwun M., Lewkowicz J., Pawlak D. (2024). Kynurenines as a novel target for the treatment of inflammatory disorders. Cells.

[11] Mor A., Tankiewicz-Kwedlo A., Krupa A., Pawlak D. (2021). Role of kynurenine pathway in oxidative stress during neurodegenerative disorders. Cells.

[12] Yano J.M., Yu K., Donaldson G.P., Shastri G.G., Ann P., Ma L. (2015). Indigenous bacteria from the gut microbiota regulate host serotonin biosynthesis. Cell.

[13] Slominski A., Pisarchik A., Zbytek B., Tobin D.J., Kauser S., Wortsman J. (2003). Functional activity of serotoninergic and melatoninergic systems expressed in the skin. J Cell Physiol.

[14] Nordlind K., Azmitia E.C., Slominski A. (2008). The skin as a mirror of the soul: exploring the possible roles of serotonin. Exp Dermatol.

[15] Huang J., Li G., Xiang J., Yin D., Chi R. (2004). Immunohistochemical study of serotonin in lesions of psoriasis. Int J Dermatol.

[16] Nordlind K., Thorslund K., Lonne-Rahm S., Mohabbati S., Berki T., Morales M. (2006). Expression of serotonergic receptors in psoriatic skin. Arch Dermatol Res.

[17] Boya B.R., Kumar P., Lee J.-H., Lee J. (2021). Diversity of the tryptophanase gene and its evolutionary implications in living organisms. Microorganisms.

[18] Russell W.R., Duncan S.H., Scobbie L., Duncan G., Cantlay L., Calder A.G. (2013). Major phenylpropanoid-derived metabolites in the human gut can arise from microbial fermentation of protein. Mol Nutr Food Res.

[19] Whitehead T.R., Price N.P., Drake H.L., Cotta M.A. (2008). Catabolic pathway for the production of skatole and indoleacetic acid by the acetogen clostridium drakei, clostridium scatologenes, and swine manure. Appl Environ Microbiol.

[20] Wlodarska M., Luo C., Kolde R., d’Hennezel E., Annand J.W., Heim C.E. (2017). Indoleacrylic acid produced by commensal peptostreptococcus species suppresses inflammation. Cell Host Microbe.

[21] Elizei S.S., Pakyari M., Ghoreishi M., Kilani R., Mahmoudi S., Ghahary A. (2018). IDO-expressing fibroblasts suppress the development of imiquimod-induced psoriasis-like dermatitis. Cell Transplant.

[22] Llamas-Velasco M., Bonay P., José Concha-Garzón M., Corvo-Villén L., Vara A., Cibrián D. (2017). Immune cells from patients with psoriasis are defective in inducing indoleamine 2,3-dioxygenase expression in response to inflammatory stimuli. Br J Dermatol.

[23] Jin L., Hu Q., Hu Y., Chen Z., Liao W. (2020). Respiratory syncytial virus infection reduces kynurenic acid production and reverses Th17/treg balance by modulating indoleamine 2,3-dioxygenase (IDO) molecules in plasmacytoid dendritic cells. Med Sci Monit: Int Med J Exp Clin Res.

[24] Hu Y., Chen Z., Zeng J., Zheng S., Sun L., Zhu L. (2020). Th17/treg imbalance is associated with reduced indoleamine 2,3 dioxygenase activity in childhood allergic asthma. Allergy Asthma Clin Immunol: Off J Can Soc Allergy Clin Immunol.

[25] Yang J., Hao T., Liu Y., Huang J., Wu W., Wu J. (2022). Th17/treg balance and indoleamine 2,3 dioxygenase activity in periodontitis-associated atherosclerotic patients. J Int Med Res.

[26] Wang M., Wang Y., Zhang M., Duan Q., Chen C., Sun Q. (2022). Kynureninase contributes to the pathogenesis of psoriasis through pro‐inflammatory effect. J Cell Physiol.

[27] Emami Z., Shobeiri S.S., Khorrami R., Haghnavaz N., Rezaee M.A., Moghadam M. (2024). Evaluation of *kynu*, *Defb2*, *camp*, and *penk* expression levels as psoriasis marker in the imiquimod‐induced psoriasis mode. Mediators Inflamm.

[28] Harden J.L., Lewis S.M., Lish S.R., Suárez-Fariñas M., Gareau D., Lentini T. (2016). The tryptophan metabolism enzyme, L-kynureninase, is a novel inflammatory factor in psoriasis and other inflammatory diseases. J Allergy Clin Immunol.

[29] Salimi Elizei S., Poormasjedi-Meibod M.-S., Wang X., Kheirandish M., Ghahary A. (2017). Kynurenic acid downregulates IL-17/1L-23 axis in vitro. Mol Cell Biochem.

[30] Schonthaler H.B., Huggenberger R., Wculek S.K., Detmar M., Wagner E.F. (2009). Systemic anti-VEGF treatment strongly reduces skin inflammation in a mouse model of psoriasis. PNAS.

[31] Cecchi M., Anceschi C., Silvano A., Coniglio M.L., Chinnici A., Magnelli L. (2024). Unveiling the role of tryptophan 2,3-dioxygenase in the angiogenic process. Pharm (Basel Switz).

[32] Mondal A., Smith C., DuHadaway J.B., Sutanto-Ward E., Prendergast G.C., Bravo-Nuevo A. (2016). IDO1 is an integral mediator of inflammatory neovascularization. EBioMedicine.

[33] Younes S.F., Bakry O.A. (2016). Immunohistochemical evaluation of role of serotonin in pathogenesis of psoriasis. J Clin Diagn Res: JCDR.

[34] Maurer M., Opitz M., Henz B.M., Paus R. (1997). The mast cell products histamine and serotonin stimulate and TNF-alpha inhibits the proliferation of murine epidermal keratinocytes in situ. J Dermatol Sci.

[35] Kim H.-E., Cho H., Ishihara A., Kim B., Kim O. (2018). Cell proliferation and migration mechanism of caffeoylserotonin and serotonin via serotonin 2B receptor in human keratinocyte HaCaT cells. BMB Rep.

[36] Zhou X., Hu Y., Liu L., Liu L., Chen H., Huang D. (2023). IL-33-mediated activation of mast cells is involved in the progression of imiquimod-induced psoriasis-like dermatitis. Cell Commun Signal: CCS.

[37] Kushnir-Sukhov N.M., Gilfillan A.M., Coleman J.W., Brown J.M., Bruening S., Toth M. (1950). 5-hydroxytryptamine induces mast cell adhesion and migration. J Immunol (Baltim Md.

[38] Cervantes-Durán C., Avalos-Viveros M., Torner L., Sánchez-Ceja S., Rodríguez-Orozco A., Martínez-Flores H. (2022). The 5‐HT_1A_ receptor agonist, 8‐OH‐DPAT, attenuates long‐lasting pain in imiquimod‐induced psoriasis in mice. Exp Dermatol.

[39] Wang M., Wang Y., Zhang M., Duan Q., Chen C., Sun Q. (2022). Kynureninase contributes to the pathogenesis of psoriasis through pro-inflammatory effect. J Cell Physiol.

[40] Kim T.-K., Kleszczynski K., Janjetovic Z., Sweatman T., Lin Z., Li W. (2013). Metabolism of melatonin and biological activity of intermediates of melatoninergic pathway in human skin cells. FASEB j: Off Publ Fed Am Soc Exp Biol.

[41] Scher J.U., Ubeda C., Artacho A., Attur M., Isaac S., Reddy S.M. (2015). Decreased bacterial diversity characterizes the altered gut microbiota in patients with psoriatic arthritis, resembling dysbiosis in inflammatory bowel disease. Arthritis Rheumatol (Hob NJ).

[42] Olejniczak-Staruch I., Ciążyńska M., Sobolewska-Sztychny D., Narbutt J., Skibińska M., Lesiak A. (2021). Alterations of the skin and gut microbiome in psoriasis and psoriatic arthritis. Int J Mol Sci.

[43] Gao J., Xu K., Liu H., Liu G., Bai M., Peng C. (2018). Impact of the gut microbiota on intestinal immunity mediated by tryptophan metabolism. Front Cell Infect Microbiol.

[44] Szelest M., Walczak K., Plech T. (2021). A new insight into the potential role of tryptophan-derived AhR ligands in skin physiological and pathological processes. Int J Mol Sci.

[45] Bieber T. (2021). AhR modulating agent for the topical therapy of plaque psoriasis. N Engl J Med.

[46] Cook C.P., Taylor M., Liu Y., Schmidt R., Sedgewick A., Kim E. (2022). A single-cell transcriptional gradient in human cutaneous memory T cells restricts Th17/Tc17 identity. Cell Rep Med.

[47] Leung D.Y., Travers J.B., Giorno R., Norris D.A., Skinner R., Aelion J. (1995). Evidence for a streptococcal superantigen-driven process in acute guttate psoriasis. J Clin Invest.

[48] Murr C., Widner B., Gerlach D., Werner-Felmayer G., Dierich M.P., Wachter H. (1997). Streptococcal erythrogenic toxins induce tryptophan degradation in human peripheral blood mononuclear cells. Int Arch Allergy Immunol.

[49] Guo L., Wu C., Song B., Jin H.-Z. (2024). Exploration of circulating metabolic signature of erythrodermic psoriasis based on LC-MS metabolomics. Exp Dermatol.

[50] Bachelez H. (2020). Pustular psoriasis: the dawn of a new era. Acta Derm Venereol.

[51] Yu N., Peng C., Chen W., Sun Z., Zheng J., Zhang S. (2021). Circulating metabolomic signature in generalized pustular psoriasis blunts monocyte hyperinflammation by triggering amino acid response. Front Immunol.

[52] Kang Z., Zhang X., Du Y., Dai S.-M. (2024). Global and regional epidemiology of psoriatic arthritis in patients with psoriasis: a comprehensive systematic analysis and modelling study. J Autoimmun.

[53] Bertazzo A., Punzi L., Bertazzolo N., Pianon M., Pozzuoli A., Costa C.V. (1999). Tryptophan catabolism in synovial fluid of various arthropathies and its relationship with inflammatory cytokines. Adv Exp Med Biol.

[54] Ospelt C. (2017). Synovial fibroblasts in 2017. RMD Open.

[55] Vandooren B., Cantaert T., ter Borg M., Noordenbos T., Kuhlman R., Gerlag D. (2008). Tumor necrosis factor alpha drives cadherin 11 expression in rheumatoid inflammation. Arthritis Rheum.

[56] Chang Y., Han P., Wang Y., Jia C., Zhang B., Zhao Y. (2022). Tryptophan 2,3-dioxygenase 2 plays a key role in regulating the activation of fibroblast-like synoviocytes in autoimmune arthritis. Br J Pharmacol.

[57] Ponikowska M., Vellone E., Czapla M., Uchmanowicz I. (2025). Challenges psoriasis and its impact on quality of life: challenges in treatment and management. Psoriasis: Targets Ther.

[58] Mrowietz U., Sümbül M., Gerdes S. (2023). Depression, a major comorbidity of psoriatic disease, is caused by metabolic inflammation. Acad Dermatol Venereol.

[59] Shen M., Cao D., Xiao Y., Kuang Y., Jing D., Li Y. (2021). Serum 5-hydroxytryptamine is related to psoriasis severity in patients with comorbid anxiety or depression. Acta Derm Venereol.

[60] Thorslund K., Svensson T., Nordlind K., Ekbom A., Fored C.M. (2013). Use of serotonin reuptake inhibitors in patients with psoriasis is associated with a decreased need for systemic psoriasis treatment: a population-based cohort study. J Intern Med.

[61] Chen Y.-H., Wang W.-M., Li I.-H., Kao H.-H., Yeh C.-B., Kao L.-T. (2021). Major depressive disorder increased risk of psoriasis: a propensity score matched cohort study. J Affect Disord.

[62] Tzeng Y.-M., Li I.-H., Kao H.-H., Shih J.-H., Yeh C.-B., Chen Y.-H. (2021). Protective effects of anti-depressants against the subsequent development of psoriasis in patients with major depressive disorder: a cohort study. J Affect Disord.

[63] Wu J.J., Kavanaugh A., Lebwohl M.G., Gniadecki R., Merola J.F. (2022). Psoriasis and metabolic syndrome: implications for the management and treatment of psoriasis. J Eur Acad Dermatol Venereol: JEADV.

[64] de Carvalho A.V.E., Romiti R., da Souza C., Paschoal R.S., Milman L.d.M., Meneghello L.P. (2016). Psoriasis comorbidities: complications and benefits of immunobiological treatment. An Bras Dermatol.

[65] Wang W., Liu L., Tian Z., Han T., Sun C., Li Y. (2021). Dietary tryptophan and the risk of metabolic syndrome: total effect and mediation effect of sleep duration. Nat Sci Sleep.

[66] Mallmann N.H., Lima E.S., Lalwani P. (2018). Dysregulation of tryptophan catabolism in metabolic syndrome. Metab Syndr Relat D.

[67] Kim H.J., Jang J., Na K., Lee E.-H., Gu H.-J., Lim Y.H. (2024). TLR7-dependent eosinophil degranulation links psoriatic skin inflammation to small intestinal inflammatory changes in mice. Exp Mol Med.

[68] De Francesco M.A., Caruso A. (2022). The gut microbiome in psoriasis and crohn’s disease: is its perturbation a common denominator for their pathogenesis?. Vaccines.

[69] Jun Y.K., Yoon H.T., Kwon S.H., Jo U.H., Kim J.E., Han Y.M. (2023). Regulation of psoriasis, colitis, and the intestinal microbiota by clusterin. Sci Rep.

[70] Nikolaus S., Schulte B., Al-Massad N., Thieme F., Schulte D.M., Bethge J. (2017). Increased tryptophan metabolism is associated with activity of inflammatory bowel diseases. Gastroenterology.

[71] Bosch S., de Meij T.G.J., de Boer N.K. (2018). Altered tryptophan levels in patients with inflammatory bowel disease owing to colonic leakage, metabolism, or malabsorption?. Gastroenterology.

[72] Lamas B., Richard M.L., Leducq V., Pham H.-P., Michel M.-L., Da Costa G. (2016). CARD9 impacts colitis by altering gut microbiota metabolism of tryptophan into aryl hydrocarbon receptor ligands. Nat Med.

[73] Bertazzo A, Punzi L, Bertazzolo N, Pianon M, Pozzuoli A, Costa CVL, et al. Tryptophan catabolism in synovial fluid of various arthropathies and its relationship with inflammatory cytokines. In: Huether G, Kochen W, Simat TJ, Steinhart H, editors. Tryptophan, Serotonin, and Melatonin, vol. 467, Boston, MA: Springer US; 1999, p. 565–70. doi: 10.1007/978-1-4615-4709-9_70. 10.1007/978-1-4615-4709-9_70 10721101

[74] Guo J., Liu Y., Guo X., Meng Y., Qi C., Zhao J. (2020). Depressive-like behaviors in mice with imiquimod-induced psoriasis. Int Immunopharmacol.

[75] Oh J.S., Seo H.S., Kim K.H., Pyo H., Chung B.C., Lee J. (2017). Urinary profiling of tryptophan and its related metabolites in patients with metabolic syndrome by liquid chromatography-electrospray ionization/mass spectrometry. Anal Bioanal Chem.

[76] Tanaka Y., Uchi H., Hashimoto-Hachiya A., Furue M. (2018). Tryptophan photoproduct FICZ upregulates IL1A, IL1B, and IL6 expression via oxidative stress in keratinocytes. Oxid Med Cell Longev.

[77] Tian C., Zhang G., Xia Z., Chen N., Yang S., Li L. (2022). Identification of triazolopyridine derivatives as a new class of AhR agonists and evaluation of anti-psoriasis effect in a mouse model. Eur J Med Chem.

[78] Eminel S., Jin N., Rostami M., Dibbert S., Mrowietz U., Suhrkamp I. (2017). Dimethyl- and monomethylfumarate regulate indoleamine 2,3-dioxygenase (IDO) activity in human immune cells. Exp Dermatol.

[79] De Simoni E., Rizzetto G., Molinelli E., Capodaglio I., Offidani A., Simonetti O. (2023). The role of diet in children with psoriasis: emerging evidence and current issues. Nutrients.

[80] Caso F., Navarini L., Carubbi F., Picchianti-Diamanti A., Chimenti M.S., Tasso M. (2020). Mediterranean diet and psoriatic arthritis activity: a multicenter cross-sectional study. Rheumatol Int.

[81] Todberg T., Egeberg A., Zachariae C., Sørensen N., Pedersen O., Skov L. (2022). Patients with psoriasis have a dysbiotic taxonomic and functional gut microbiota. Br J Dermatol.

[82] Buhaș M.C., Candrea R., Gavrilaș L.I., Miere D., Tătaru A., Boca A. (2023). Transforming psoriasis care: probiotics and prebiotics as novel therapeutic approaches. Int J Mol Sci.

[83] Stec A., Sikora M., Maciejewska M., Paralusz-Stec K., Michalska M., Sikorska E. (2023). Bacterial metabolites: a link between gut microbiota and dermatological diseases. Int J Mol Sci.

[84] Navarro-López V., Martínez-Andrés A., Ramírez-Boscá A., Ruzafa-Costas B., Núñez-Delegido E., Carrión-Gutiérrez M. (2019). Efficacy and safety of oral administration of a mixture of probiotic strains in patients with psoriasis: a randomized clinical trial. Acta Derm Venereol.

[85] Modulation of gut propionate and intestinal mucosal protection by bifidobacterium longum: mitigating methotrexate side effects without compromising the efficacy of psoriasis therapy. Int Immunopharmacol 2025;149:114196. doi: 10.1016/j.intimp.2025.114196. 10.1016/j.intimp.2025.114196 39904035

[86] Suriano E.S., Souza M.D.M., Kobata C.M., Santos F.H.Y., Mimica M.J. (2023). Efficacy of an adjuvant lactobacillus rhamnosus formula in improving skin lesions as assessed by PASI in patients with plaque psoriasis from a university-affiliated, tertiary-referral hospital in são paulo (brazil): a parallel, double-blind, randomized clinical trial. Arch Dermatol Res.

[87] Allison D.J., Nederveen J.P., Snijders T., Bell K.E., Kumbhare D., Phillips S.M. (2019). Exercise training impacts skeletal muscle gene expression related to the kynurenine pathway. Am J Physiol Cell Physiol.

[88] Sivasami P., Elkins C., Diaz-Saldana P.P., Goss K., Peng A., Hamersky M. (2023). Obesity-induced dysregulation of skin-resident PPARγ+ treg cells promotes IL-17A-mediated psoriatic inflammation. Immunity.

[89] Sonomoto K., Song R., Eriksson D., Hahn A.M., Meng X., Lyu P. (2023). High-fat-diet-associated intestinal microbiota exacerbates psoriasis-like inflammation by enhancing systemic γδ T cell IL-17 production. Cell Rep.

[90] Jensen P., Christensen R., Zachariae C., Geiker N.R., Schaadt B.K., Stender S. (2016). Long-term effects of weight reduction on the severity of psoriasis in a cohort derived from a randomized trial: a prospective observational follow-up study. Am J Clin Nutr.

[91] L N, A C, S C, A P, M P, A L, et al. Diet and physical exercise in psoriasis: a randomized controlled trial. Br J Dermatol 2014;170. doi: 10.1111/bjd.12735. 10.1111/bjd.12735 24641585

[92] Jensen P., Zachariae C., Christensen R., Geiker N.R.W., Schaadt B.K., Stender S. (2014). Effect of weight loss on the cardiovascular risk profile of obese patients with psoriasis. Acta Derm Venereol.

[93] Betari N., Teigen K., Sahlholm K., Haavik J. (2021). Synthetic corticosteroids as tryptophan hydroxylase stabilizers. Future Med Chem.

[94] Janjetovic Z., Nahmias Z.P., Hanna S., Jarrett S.G., Kim T.-K., Reiter R.J. (2014). Melatonin and its metabolites ameliorate ultraviolet B-induced damage in human epidermal keratinocytes. J Pineal Res.

[95] Yu B., Becnel J., Zerfaoui M., Rohatgi R., Boulares A.H., Nichols C.D. (2008). Serotonin 5-hydroxytryptamine(2A) receptor activation suppresses tumor necrosis factor-alpha-induced inflammation with extraordinary potency. J Pharmacol Exp Ther.

